# Association Between Cigarette and Bidi Purchase Behavior (Loose vs Pack) and Health Warning Label Exposure: Findings From the Tobacco Control Policy India Survey and In-Depth Interviews With People Who Smoke

**DOI:** 10.2196/63193

**Published:** 2024-09-25

**Authors:** Mayank Sakhuja, Daniela B Friedman, Mark M Macauda, James R Hebert, Mangesh S Pednekar, Prakash C Gupta, Geoffrey T Fong, James F Thrasher

**Affiliations:** 1 UNC Lineberger Comprehensive Cancer Center University of North Carolina at Chapel Hill Chapel Hill, NC United States; 2 Department of Health Promotion, Education and Behavior Arnold School of Public Health University of South Carolina Columbia, SC United States; 3 Department of Epidemiology and Biostatistics and Cancer Prevention and Control Program Arnold School of Public Health University of South Carolina Columbia, SC United States; 4 Healis Sekhsaria Institute for Public Health Navi Mumbai India; 5 Department of Psychology University of Waterloo Waterloo, ON Canada; 6 School of Public Health Sciences University of Waterloo Waterloo, ON Canada; 7 Ontario Institute for Cancer Research Toronto, ON Canada

**Keywords:** loose cigarettes, singles, health warning labels, tobacco control, India, mixed methods, purchase behavior, tobacco users, cigarette, qualitative research, thematic analysis, prevention, health promotion, public health

## Abstract

**Background:**

The sale of loose cigarettes or bidis can undermine the purpose of requiring health warning labels (HWLs) on cigarette packs and bidi bundles by diminishing their visibility and legibility.

**Objective:**

This mixed-methods study aims to examine the association between purchase behavior (loose vs pack or bundle), HWL exposure, and responses to HWLs among Indian adults who smoke.

**Methods:**

Data were analyzed from the 2018-2019 India Tobacco Control Policy Survey and from 28 in-depth interviews conducted with Indian adults who smoked in 2022. The Tobacco Control Policy Survey sample included tobacco users who bought cigarettes (n=643) or bidis (n=730), either loose or in packs or bundles at their last purchase. Ordinal regression models were fit separately for cigarettes and bidis, whereby HWL variables (noticing HWLs, reading and looking closely at HWLs, forgoing a cigarette or bidi because of HWLs, thinking about health risks of smoking, and thinking about quitting smoking cigarettes or bidis because of HWLs) were regressed on last purchase (loose vs packs or bundles). In-depth interviews with participants from Delhi and Mumbai who purchased loose cigarettes in the last month were conducted, and thematic analysis was used to analyze the interview data.

**Results:**

Survey findings indicated that about 74.3% (478/643) of cigarette users and 11.8% (86/730) of bidi users reported having bought loose sticks at their last purchase. Those who purchased loose cigarettes (vs packs) noticed HWLs less often (estimate –0.830, 95% CI –1.197 to –0.463, *P*<.001), whereas those who purchased loose bidis (vs bundles) read and looked closely at HWLs (estimate 0.646, 95% CI 0.013-1.279, *P*=.046), thought about the harms of bidi smoking (estimate 1.200, 95% CI 0.597-1.802, *P*<.001), and thought about quitting bidi smoking (estimate 0.871, 95% CI 0.282-1.461, *P*=.004) more often. Interview findings indicated lower exposure to HWLs among those who purchased loose cigarettes, primarily due to vendors distributing loose cigarettes without showing the original cigarette pack, storing them in separate containers, and consumers’ preference for foreign-made cigarette brands, which often lack HWLs. While participants were generally aware of the contents of HWLs, many deliberately avoided them when purchasing loose cigarettes. In addition, they believed that loose cigarette purchases reduced the HWLs’ potential to deliver consistent reminders about the harmful effects of cigarette smoking due to reduced exposure, an effect more common among those who purchased packs. Participants also noted that vendors, especially small ones, did not display statutory health warnings at their point of sale, further limiting exposure to warning messages.

**Conclusions:**

Survey and interview findings indicated that those who purchased loose cigarettes noticed HWLs less often. Loose purchases likely decrease the frequency of exposure to HWLs’ reminders about the harmful effects of smoking, potentially reducing the effectiveness of HWLs.

## Introduction

### Background

Tobacco use is one of the top causes of preventable deaths in India, accounting for 1.3 million deaths every year [1,2]. Pictorial health warning labels (HWLs) on all tobacco products are a promising strategy for reducing the mortality and morbidity associated with tobacco use. Article 11 of the World Health Organization Framework Convention on Tobacco Control recommends that every country should mandate that at least 50% area of tobacco product packages should depict large, clear, and rotating HWLs that convey the harmful effects of tobacco use [3]. Since 2014, the Cigarettes and Other Tobacco Products Act (COTPA) in India requires that at least 85% of the total area on the front and back of tobacco product packs must have pictorial HWLs. Visual examples of the most recent packs of tobacco products with their HWLs in India are available on the TPackSS website [4]. In addition, tobacco vendors in India are required to put up a statutory warning stating that selling tobacco products to minors is prohibited, along with a pictorial health warning depicting oral cancer [5,6].

HWLs are a low-to-no-cost strategy and have a broad, population-level reach for communicating health information to people who smoke and those who do not [7,8]. Cigarette packages are an effective medium for reaching individuals who purchase cigarette packs because they are potentially exposed to HWLs every time they reach for a cigarette [9,10]. Moreover, exposure to HWLs is also associated with higher-risk perceptions, increased knowledge and awareness regarding harms from tobacco use, and has a greater potential to enable individuals to quit smoking [11-14].

The availability of loose cigarettes potentially reduces the effectiveness of HWLs as individuals who use loose cigarettes do not carry them around in the packaging on which HWLs are printed [15-18]. The sale of loose cigarettes is widespread in many low- and middle-income countries, including the Philippines, Mexico, Guatemala, Vietnam, Uruguay, Thailand, Brazil, Bangladesh, and India [19-21]. This practice is also prevalent in lower socioeconomic neighborhoods of high-income countries, such as the United States [15,22-24]. In addition, despite legal bans, the prevalence of loose cigarette sales has continued to rise over time in some low- and middle-income countries, such as Mexico [25]. According to the 2016-2017 Global Adult Tobacco Survey in India, about 67% of people who smoke cigarettes and 17% of people who smoke bidis purchased them loose at their last purchase [26]. Lal et al [27] found that nearly 75% of all cigarettes sold in India were sold loose.

### This Study

To the best of our knowledge, no study has evaluated whether the purchase of loose cigarettes and bidis reduces the effectiveness of HWLs. This mixed-methods study examines associations between cigarette and bidi purchase behavior and self-reported responses to HWLs among Indian adults who smoke.

## Methods

### Data Sources

This paper uses data from 2 sources. First, data from the 2018-2019 Tobacco Control Policy (TCP) India Survey were analyzed [28]. These data were collected from the rural and urban areas of 4 Indian states (West Bengal, Maharashtra, Madhya Pradesh, and Bihar) using multistage cluster sampling of households to obtain representative samples at state level. Respondents in the 2018-2019 wave of TCP data collection included people who used smoked tobacco, smokeless tobacco, mixed-tobacco, people who have quit, and nonusers [28]. Second, as part of a qualitative project focused on the implementation and enforcement of the ban on the sale of loose cigarettes and bidis, data from in-depth interviews of people who smoked were also used in this study. Individuals were recruited using a standardized protocol and interviewed in Hindi (a local Indian language) in 2 Indian cities, Delhi and Mumbai. Users who purchased loose cigarettes or bidis were recruited at the points of sale across different regions in both cities and were interviewed outside, near the point of sale where they were approached. Snowball sampling was also used where interviewed individuals were asked to recommend other potential participants [29] who were interviewed via Zoom (Zoom Video Communications Inc). Detailed recruitment and interview protocol using the COREQ (Consolidated Criteria for Reporting Qualitative Research) checklist [30] has been published elsewhere [31]. We used the STROBE (Strengthening the Reporting of Observational Studies in Epidemiology) cross-sectional checklist [32] to guide this current work.

### Study Sample

A total of 1373 respondents from the TCP survey data were included in the analytic sample, who reported buying either loose or packaged cigarettes or bundled bidis at their last purchase (n=643, cigarette users and n=730, bidi users). Qualitative interviews were conducted with 13 individuals in Mumbai (where the ban on the sale of loose cigarettes was implemented) and 15 individuals in Delhi (where the ban was not implemented), all of whom reported purchasing loose cigarettes at least once in the last 30 days.

### Measures

For analysis of the data obtained from the TCP survey, the main independent variable was purchasing behavior, determined by responses to whether participants had purchased cigarettes or bidis loose or in packs or bundles at their last purchase. Dependent variables included variables related to the effectiveness of HWLs, including noticing HWLs (recoded as 0=never and don’t know, 1=once in a while, 2=often, and 3=whenever I smoke cigarettes), reading or looking closely at HWLs (recoded as 0=never and don’t know, 1=rarely, and 2=regularly, often, and once in a while), forgoing a cigarette or bidi because of HWLs (recoded as 0=never and don’t know, 1=a couple of times, and 2=many times and once in a while), thinking about the health risks of smoking because of HWLs (recoded as 0=not at all and do not know, 1=a little, and 2=a lot), and thinking about quitting smoking cigarettes or bidis because of HWLs (recoded as 0=not at all and do not know, 1=a little, and 2=a lot). Categories for all the dependent variables were recoded to maintain adequate sample sizes for the analysis. Sociodemographic and tobacco use variables included sex (male or female), age (18-30 years, 31-40 years, 41-50 years, and 51 years and older), education level (low, moderate, or high), intentions to quit in the next 6 months (yes or no), and smoking frequency (daily or nondaily).

Qualitative interview questions focused on assessing individuals’ awareness of and knowledge about the ban on the sale of loose cigarettes and bidis, as well as how the policy had and could impact their smoking behavior. Individuals were also asked to describe their most recent experience of visiting a tobacco vendor to purchase loose cigarettes or bidis for which they were probed about how often they noticed HWLs at the vendor’s establishment and on cigarette packs at the time of purchase. Their perceptions regarding exposure to HWLs have been analyzed in this study.

### Data Analysis

We performed descriptive analysis (crosstabulations, frequencies, and percentages) between purchase behavior (loose vs pack or bundle) and HWL variables (noticing HWLs, reading or looking closely at HWLs, forgoing a cigarette or bidi because of HWLs, thinking about the health risks of smoking because of HWLs, and thinking about quitting smoking cigarettes or bidis because of HWLs). We conducted ordinal regression analysis and models were estimated separately for cigarette and bidis, whereby HWL variables were regressed on purchase behavior. All models were adjusted for age, sex, education, neighborhood, smoking frequency, and intentions to quit. Regression analysis was performed using SPSS (version 28; IBM Corp) [33].

Reflexive thematic analysis [34] was performed for the qualitative interviews and all data were organized using the NVivo qualitative software (Lumivero) [35]. Transcripts were prepared by translating interview recordings from Hindi to English. A preliminary codebook was developed to guide the analysis. Three authors independently coded one transcript to further refine the codebook. Line-by-line analysis was conducted on each additional transcript and new codes were added to the existing codebook [36]. Codes were then organized and grouped into common and meaningful themes for interpretation [37].

### Ethical Considerations

The TCP India Survey protocols and all materials, including the survey questionnaires, were approved by the Research Ethics Board, University of Waterloo, Canada (REB#22140/31086) and the Healis Sekhsaria Institute for Public Health Institutional Ethics Committee, India (IRB00007340). The qualitative study was approved by the institutional review board of the University of South Carolina (Pro00120549) and the institutional ethics committee of Healis Sekhsaria Institute for Public Health (IRB00007340; FWA00019699).

## Results

### Sample Characteristics

#### TCP Survey Respondents

The sample of 1373 individuals consisted mainly of male participants (n=1350, 98.3%), those from urban neighborhoods (n=980, 71.4%), and those with low educational attainment (n=846, 62%). About 72.3% (992/1373) were exclusive users of smoked tobacco, 81.3% (1113/1373) smoked daily, 81% (1099/1373) had never attempted to quit cigarette or bidi smoking, and 94% (1257/1373) had no intentions to quit cigarette or bidi smoking in the next 6 months. About three-quarters (478/643, 74.3%) of cigarette users reported purchasing loose cigarettes at their last purchase, whereas only 11.8% (86/730) of bidi users purchased loose bidis at their last purchase.

#### Qualitative Interview Participants

The mean age of the 28 individuals (n=15, 54% from Delhi and n=13, 46% from Mumbai) interviewed was 26.4 (SD 6.2) years. All participants had purchased cigarettes (vs bidis) at their last purchase and 86% (24/28) purchased them in loose. About 61% (17/28) smoked daily, 89% (25/28) were exclusive smoked-tobacco users, and 71% (20/28) had no intentions to quit smoking in the next 6 months. Most were male respondents (19/28, 68%) and had high (at least an undergraduate degree) educational attainment (18/28, 64%).

### Survey Findings

#### Association Between HWL Responses and Purchase Behavior

About 15% (93/627) of the cigarette users (vs 39/511, 7.6% of the bidi users) reported never noticing HWLs on cigarette (bidi) packages. About 34% (182/534) of the cigarette users (vs 194/471, 41.2% of the bidi users) reported never reading or looking closely at HWLs on cigarette (or bidi) packages and 81.5% (510/626) of the cigarette users (vs 374/511, 73.2% of the bidi users) reported that the HWLs never stopped them from having a cigarette (or bidi) when they were about to have one. Crosstabulations between HWL responses and purchase behavior (pack vs loose) for both cigarettes and bidis are presented in Table 1.

After adjustment for covariates, individuals who purchased loose cigarettes at their last purchase (vs packs) noticed HWLs less often. Other HWL responses did not have any significant (all *P*>.05) association with purchase behavior (Table 2).

In adjusted models, no association was found between purchase behavior and noticing HWLs on bidi bundles; however, those who purchased loose bidi at their last purchase (vs bundle) read and looked closely at HWLs and thought about the health risks of bidi smoking and quitting bidi smoking more often due to the HWLs (Table 3).

**Table 1:** Crosstabulations between health warning label (HWL) responses and purchase behavior for cigarettes and bidis among Indian adults who smoke.

Measure			Cigarette users, n (%)				Bidi users, n (%)		
			Pack		Loose		Bundle		Loose
**In the last 30 days, how often have you noticed HWLs on cigarette or bidi packages?**									
	Never/don’t know	18/162 (11.1)		75/465 (16.2)		34/465 (7.3)		5/46 (10.9)	
	Once in a while	17/162 (10.5)		105/465 (22.6)		67/465 (14.4)		11/46 (23.9)	
	Often	85/162 (52.5)		229/465 (49.2)		268/465 (57.6)		24/46 (52.1)	
	Whenever I smoke cigarettes and bidis	42/162 (25.9)		56/465 (12)		96/465 (20.6)		6/46 (13)	
**In the last 30 days, how often have you read and looked closely at HWLs on cigarette or bidi packages?**									
	Never/don’t know	47/144 (32.6)		135/390 (34.6)		186/430 (43.3)		8/41 (19.5)	
	Rarely	49/144 (34)		127/390 (32.6)		89/430 (20.7)		14/41 (34.1)	
	Regularly and often and once in a while	48/144 (33.3)		128/390 (32.8)		155/430 (36)		19/41 (46.3)	
**In the last 30 days, have the HWLs stopped you from having a cigarette or bidi when you were about to have one?**									
	Never/don’t know	130/162 (80.2)		380/464 (81.9)		345/465 (74.2)		29/46 (63)	
	A couple of times	19/162 (11.7)		56/464 (12.1)		50/465 (10.8)		11/46 (23.9)	
	Many times and once in a while	13/162 (8)		28/464 (6)		70/465 (15.1)		6/46 (13)	
**To what extent does the HWL make you more likely to think about the health risks of smoking?**									
	Not at all/don’t know	83/162 (51.2)		196/458 (42.8)		184/458 (40.2)		8/46 (17.4)	
	A little	60/162 (37)		191/458 (41.7)		209/458 (45.6)		24/46 (52.2)	
	A lot	19/162 (11.7)		71/458 (15.5)		65/458 (14.2)		14/46 (30.4)	
**To what extent does the HWL make you more likely to quit smoking?**									
	Not at all/don’t know	96/161 (59.6)		226/459 (49.2)		218/456 (47.8)		13/46 (28.3)	
	A little	51/161 (31.7)		153/459 (33.3)		172/456 (37.7)		20/46 (43.5)	
	A lot	14/161 (8.7)		80/459 (17.4)		66/456 (14.5)		13/46 (28.3)	

**Table 2:** Adjusted ordinal regression models for health warning label (HWL) responses and cigarette purchase behavior among Indian adults who smoke.

Estimates for loose vs pack (reference) purchase^a^	Notice HWL (n=605)	Read and look closely at HWL (n=516)	Forgo a cigarette because of HWL (n=604)	Think about the health risks of cigarettes because of HWL (n=599)	Think about quitting cigarettes because of HWL (n=598)
Estimate (95% CI)	–0.830 (–1.197 to –0.463)	0.052 (–0.324 to 0.429)	–0.179 (–0.666 to 0.309)	0.247 (–0.118 to 0.613)	0.348 (–0.027 to 0.724)
*P* value	<.001^b^	.79	.47	.18	.07

^a^Estimates were for purchasing loose versus pack (reference) cigarettes at the last purchase. All models were adjusted for sex, age, education level, neighborhood, quit intentions, and smoking frequency.

^b^*P*<.001.

**Table 3:** Adjusted ordinal regression models for health warning label (HWL) responses and bidi purchase behavior among Indian adults who smoke.

Variable	Notice HWL (n=501)	Read and look closely at HWL (n=461)	Forgo a bidi because of HWL (n=501)	Think about the health risks of bidis because of HWL (n=494)	Think about quitting bidis because of HWL (n=492)
Estimate^a^ (95% CI)	–0.438 (–1.035 to 0.160)	0.646 (0.013-1.279)	0.541 (–0.130 to 1.211)	1.200 (0.597-1.802)	0.871 (0.282-1.461)
*P* value	.15	.046^b^	.11	<.001^c^	.004^b^

^a^Estimates were for purchasing loose versus bundle (reference) bidis at the last purchase. All models were adjusted for sex, age, education level, neighborhood, quit intentions, and smoking frequency.

^b^*P*<.05.

^c^*P*<.001.

### Qualitative Findings

#### Results From Both Cities

In-depth interviews were conducted with people who had recently purchased loose cigarettes in 2 Indian states, Mumbai and Delhi. Even though the ban on the sale of loose cigarettes in Mumbai was implemented at the time of data collection, compliance with and awareness of the ban was poor, and study participants reported no issues in accessing loose cigarettes in the city. We, therefore, present the results of both the cities together. In the following sections, we have presented participants’ perceptions about noticing HWLs on cigarette packs at the point of sale at the time of purchasing loose cigarettes.

#### Noticing HWLs at the Time of Purchase

Interview participants were asked if they noticed HWLs at the time of purchasing loose cigarettes. Participants reported that they did not frequently notice HWLs on cigarette packs as the vendor directly handed them out single cigarette sticks rather than showing them the cigarette pack:

I would say, not very frequently because what happens is that the shop owner gives us the cigarette in our hands if I am buying one or two cigarettes. So, we don’t get to touch the cigarette packet, so we don’t get to see the label over it also.

Male, aged 25 years, high education level

It was reported that loose cigarettes were usually kept in separate containers which the buyers did not have access to, and as a result, did not get to notice HWLs on cigarette packs at the time of purchase:

No, I cannot see at that time. Obviously, if someone is buying one [cigarette] then he will not see the box. Sometimes, it is there down in his drawers. So, he takes it directly from there and gives it to me. So, I cannot see the box.

Male, aged 23 years, high education level

He actually takes it out from the box and then gives it to us. He doesn’t show that he is taking it out from the box in front of us. Usually, they have it in a container as such.

Female, aged 26 years, high education level

Participants reported being aware that cigarette packs included pictorial HWLs. Even though the vendor opened the cigarette pack in front of the buyers to hand out loose cigarettes, buyers themselves tended not to notice HWLs on the cigarette packs at the time of purchase:

I think...I will tell him to give me an Indie Mint, so he just takes one packet, takes it out, and gives it to me. I don’t really notice what’s written on the packet. I know what’s written on the packet. But I don’t really notice the health warning that’s on them.

Female, aged 21 years, high education level

However, some participants also mentioned noticing HWLs on cigarette packs stating that the vendor took out the loose cigarette from the pack in front of them because of which they were able to notice both pictorial and text warnings:

Yeah, I mean, he pulled it out from a box. So obviously I saw that, like smoking kills and that graphic image of I don’t know...I don’t even know what.

Female, aged 22 years, high education level

Exposure to HWLs was not just limited to cigarette packs. Participants also reported noticing warning messages at and around the point of sale from where they purchased cigarettes. Some participants noticed statutory warning messages through warning boards that were put up at the vendor’s establishment which vendors were required to display as per the tobacco control law:

It is always visible, because if you go to a proper pan [tobacco] shop, they have this huge signboard, so suppose cigarette company is sponsoring him or something, they will put up the ad, there will be this statutory warning and stuff.

Female, aged 33 years, high education level

In addition to the warnings that the vendors were required to put up at their establishment, few cigarette users reported seeing pictorial HWLs on cigarette packs that were on display inside the vendor’s establishment and through the empty cigarette packs that were thrown out around the establishment:

Yes [could see health warnings], because he [the vendor] keeps everything on display, so you can clearly see that.

Female, aged 33 years, high education level

The used boxes that were empty, that were thrown out. They had those images.

Female, aged 26 years, high education level

Not all tobacco vendors put up statutory warnings inside their establishments or are able to display cigarette packs because of limited space inside their establishment. Participants who purchased cigarettes from small vendors, such as street vendors or small tobacco kiosks usually did not get exposed to any health messages as those vendors did not put up any statutory warnings:

No, street vendors do not put any warnings on their setup. Because late night vendors do not have at all, and not only this type, but then there are other people also who sell in the midnight like boost, coffee, and tea on their cycles, so they have this milk and tea in the thermos but then they carry cigarette packets in plastic bag.

Female, aged 33 years, high education level

So no, they have not displayed the packets anywhere because it is also a tea shop, so they keep it in a drawer. It is not visible.

Female, aged 34 years, high education level

Participants mentioned that noticing HWLs on cigarette packs also depended on the number of loose cigarettes being purchased by the buyer. Those who bought multiple loose cigarettes did notice HWLs on cigarette packs as they tended to ask for an empty cigarette box in which they could keep their loose cigarettes because of which they noticed HWLs as well:

Sometimes he even gives six cigarettes in the box. We cannot keep it just like that. Cigarettes are very delicate. They break. So, he puts it in a box and gives it. It is obvious. I ask for six cigarettes, and he puts them in the box and gives it to me. It is written on the box that tobacco causes cancer.

Male, aged 23 years, high education level

However, participants who preferred foreign-made cigarette brands did not notice HWLs on cigarette packs because not all foreign brands (eg, Esse Lights) have mandatory pictorial warnings on their cigarette packs:

Actually, the cigarettes that I smoke don’t have any pictorial representation. It (pictorial warning) is not there on the Esse Lights box. They don’t have that because it’s a foreign brand. They have it [text warning] written, like...not in a very big way. It’s written in a very tiny font, I should say.

Female, aged 26 years, high education level

He took the box in front of me, but the picture was not there, so it was not seen clearly.

Male, aged 28 years, high education level

Participants reported that to reduce exposure to health warning messages, tobacco vendors tried different tactics so that buyers could not read warning messages. They mentioned that the vendors would tamper with the warnings, put up warnings of smaller sizes, or change the lighting around it so that it was not clearly visible:

It’s like...so the thing is everybody reads it...but it is so ignorant in that way that...they are placing the warnings with the tobacco company’s advertisement...like a big image of a cigarette is advertised and below that it is written “tobacco kills,” nobody is going to focus on that, right!

Male, aged 28 years, high education level

Shopkeepers do hang them [warnings] but it is so dull, means where the warning is mentioned, they won’t put a light next to it, so that people cannot see the warning...I have never noticed it, even if I had, nobody put warnings of big enough size.

Male, aged 25 years, moderate education level

But nobody notice that, they either blur the warning or make it dark, so that no one notices them. There is no sense of putting the warning, people do not pay attention to them.

Male, aged 25 years, moderate education level

#### Reactions to Getting Exposed to HWLs on Cigarette Packs

Participants described behaviors to avoid exposure to HWLs. They mentioned that they tended to avoid seeing or noticing HWLs because they did get exposed to them frequently and described that they were habituated to the warnings:

No, it does not really always happen that I get to view pictorial warnings, because like I said most of the times I smoke because I am stressed or I really want to easen up. That’s when I smoke. So, now I’m just used to seeing-seeing all of those things. So, like memory chooses to avoid it, rather than you know, keeping on watching it every now and then. So, that’s what it is.

Male, aged 27 years, high education level

I think a part of us just becomes ignorant and we just start ignoring those because of course they are awful to look at.

Female, aged 26 years, high education level

#### Perceived Effectiveness of HWLs

One participant who bought a cigarette pack at their last purchase mentioned that the labels elicited negative affect which made them think of the harmful effects of cigarette smoking:

It just makes you think that, you know, what if...of course what they show is like very extreme but like at times they make me think that “can this really impact this bad?” But then I think, then we just end up ignoring that. And I often try and flip the pack so that I don’t see it but on the other side also it is there. So, I just put it in the bag. I don’t just carry the entire box everywhere, every time. It is generally in the bag, I don’t really see it all the time. So I think I have just had that ignored...I think we are very self or we are very selective for that matter, I think all of us women we take in what we really want to hear or see and we tend to ignore the rest. So, I think that’s something that I do now. But the thought that I told you has crossed my mind a couple of times but yeah.

Female, aged 26 years, high education level

Participants revealed that HWLs on cigarette packs helped keep health messages vividly in the minds of the people who smoke. Even though the purchase of loose cigarettes reduces the frequency of noticing HWLs, people who purchased loose cigarettes did get exposed to them at least once, which kept the health message alive in their minds:

Yes, everyone knows, all get exposed to the warnings on the cigarette packet. If I have seen once, that there is a picture depicting cancer, that if we smoke cigarette, we can get cancer, now even if I go to purchase cigarettes 10 times and not get exposed to the warnings those 10 times, but I would still know that I can get cancer if I smoke. So, out of 10 times, people do get exposed at least once, that they can get cancer from it.

Male, aged 19 years, low education level

However, they also believed that the purchase of loose cigarettes decreased the likelihood of HWLs delivering constant reminders about the harmful effects of cigarette smoking owing to the reduced exposure and decreased the likelihood of generating stronger beliefs about the seriousness of the threat from smoking:

If somebody has a box with him or her, if they are carrying the box with them, then that will act as a constant reminder to them. They would keep looking at it and think it’s very ugly. Like if you see that poster (with pictorial warnings), it’s very...you tend to feel disgusting when you see that. And then you tend to think that the same thing can happen with us as well. In loose cigarettes, yes, there is a difference. In that case, you will see such a warning maybe just once max to max, and that too if the vendor takes it out from the cigarette pack in front of you.

Male, aged 22 years, moderate education level

## Discussion

### Principal Findings

Findings for cigarette users in both the survey and the qualitative interviews were consistent in indicating that the purchase of loose cigarettes reduces exposure to HWLs. Where survey findings highlighted that those who purchased loose cigarettes noticed HWLs on cigarette packs less often, interview findings explained the specific mechanisms through which the exposure is reduced, such as packs not being visible, avoidance behavior, tobacco vendors not putting up statutory warnings at the point of sale, and availability of foreign-made cigarettes that do not carry the required HWLs on their cigarette packs.

To the best of our knowledge, this is the first study to provide evidence which proves that exposure to HWLs is lower among those who purchase loose cigarettes compared with packed cigarettes, while also explaining the specific mechanisms through which the exposure gets reduced. However, survey results did not find any significant associations between purchase behavior and other indicators of HWL effectiveness, such as forgoing a cigarette, thinking about the health risks of cigarette smoking, and quitting because of HWLs. Hence, it is possible that loose cigarette users, like pack purchasers, are likely to have been exposed to HWLs on multiple occasions and may have thought about accumulated exposures over a longer period of time. As a result of these exposures, they may be equally aware as pack purchasers of the contents of HWLs and the health risks of cigarette smoking. They may have thought about these accumulated exposures, even though our questions about HWLs asked about responses over the prior month. In addition, survey respondents were asked to respond only about their last purchase, and it may be possible that those who reported buying loose cigarettes at their last purchase might have bought cigarette packs in the past. Future studies should further explore this possibility by analyzing exclusive loose cigarette users as a separate group, which our study did not query.

For those who purchased loose bidis (vs bidi bundles) at their last purchase, we found that they more often read and looked closely at HWLs, and thought about the health risks of bidi smoking and quitting bidi because of HWLs on bidi packs. That could be possible as people who purchase loose could be more interested in quitting and could have a stronger response to HWLs even though they may be less often exposed. In contrast, lower quitting intentions among those who purchased bidi bundles could be attributed to poor compliance with the HWL law for bidis in India. A recent study by Saraf et al [38] that examined the extent of compliance of HWLs on bidis in India found that none of the bidi packs were compliant with the law requirements. Noncompliance issues pertained to nonstandardized packaging, incomplete HWLs, poorly printed HWLs, and old HWLs [38]. Consistent with that, another study based in India found that about 94% of bidis were not compliant with the COTPA sections 7, 8, and 9 meaning they either did not have warnings on both sides of the pack, did not meet the minimum stipulated height and width requirement, or the language of the text warning was different than that of the pack [39]. There is a need to use an in-depth mixed-methods approach involving exclusive loose- and bundled-bidi users to further explore how bidi purchases impact HWL effectiveness.

There has been an increasing prevalence of foreign-made cigarettes, which are sold illegally in India and likely not only reduce HWL exposures but also violate the provisions of the COTPA. Qualitative findings from our study found that those who were users of foreign-made cigarettes never noticed HWLs as packs of foreign-made cigarettes do not have HWLs on them. These findings are consistent with other studies in India. Chahar et al [6] found that there was poor compliance with sections 7, 8, and 9 of COTPA among foreign cigarette brands. Only 11% of the foreign-made cigarettes depicted pictorial HWLs on their cigarette packs, significantly reducing HWL exposure among those who purchase foreign-made cigarettes [6].

In addition to noticing HWLs on cigarette packs, interviewed participants also described noticing health warning messages at the point of sale because vendors were mandated to put up statutory health warnings at the entrance of their establishments. Some participants reported seeing those statutory warnings or pictorial HWLs on cigarette packs displayed inside big tobacco stores. Because compliance with depicting oral cancer in the statutory warning is poor, it may make the warning easier to ignore. Pictorial HWLs generate stronger negative affect and attitudes compared with text-only warnings [40]. However, not every point of sale or vendor displays the statutory warning in the prescribed format, and, even if they do, it may be masked. Per the 2016-2017 Global Adult Tobacco Survey in India, about 48% of people who currently smoke, purchased their last cigarette from small tobacco kiosks or street vendors [26]. Findings from our study indicate that vendors like street vendors and small tobacco kiosks did not put up any statutory warnings at their establishments nor did they display cigarette packs with HWLs clearly visible, thus reducing exposure to health warning messages through HWLs and statutory warnings. Our finding is consistent with the prior literature highlighting a substantial percentage of tobacco vendors displaying advertisement boards without health warnings [41] and higher noncompliance to the presence of health warnings especially among small vendors [42].

Even though this paper primarily focused on exposure to HWLs, exposure is also considered as a gateway to other HWL effects. For example, exposure is necessary for promoting a message process that leads to greater knowledge and risk perceptions about cigarette smoking [43]. Indeed, those who purchase loose cigarettes (compared with packs) have been found to have lower knowledge about the health effects of smoking [44]. With the plans to update HWL content in India [45], reduced HWL exposure because of the loose sale of cigarettes could deter the dissemination of new knowledge.

Our interviews with loose cigarette users described an avoidance behavior in which they ignored the health warnings because they were already exposed to them and did not like seeing them. Literature on warning avoidance is inconsistent. The Extended Parallel Process Model describes avoidance behavior as a defensive reaction that deters message processing [46], which is consistent with interview participants’ descriptions of avoiding health warnings to rationalize and continue smoking. However, larger surveys have found that warning-avoidance behaviors are either unassociated [47,48] or positively associated with cessation attempts [49-51]. More research is needed, specifically in India, to understand how warning avoidance is associated with cessation.

Finally, the purpose of HWLs is to generate firmer beliefs about the harms of smoking and aid in promoting smoking cessation [52-54]. Randomized controlled trials have found that HWLs are associated with forgoing cigarettes, intentions to quit, negative emotional reactions such as fear, thinking about the warning message, and the harms of smoking [13]. A meta-analysis found that HWLs eliciting affective and cognitive reactions are very effective in motivating individuals to quit cigarette smoking [14]. In line with the literature, a participant from our study, who purchased a cigarette pack at their last purchase described that they experienced negative reactions (felt awful) on seeing HWLs on cigarette packs and that the HWLs made them think about the harms of smoking. Participants who bought loose cigarettes at their last purchase admitted that this was not true for loose cigarette purchasers. They further described that HWLs acted as a constant reminder about the harms of smoking, elicited negative reactions of feeling disgusted, and made the users think about the seriousness of the threat from smoking for those who purchased packs. Study findings reveal that even though interview participants were generally aware of the contents of HWLs, those who purchase loose cigarettes were less often exposed to the constant reminders from HWLs, which can be important for strengthening and making more accessible beliefs about the harmful effects of cigarette smoking. Findings suggest that loose cigarette users had weaker beliefs about the seriousness of the threat from smoking compared with pack purchasers. This implies that the availability of loose cigarettes potentially reduces the overall effectiveness of HWLs.

### Limitations

This study has some limitations. Survey findings cannot be generalized to the entire country as the survey was conducted in only 4 Indian states and is not nationally representative. In addition, the cross-sectional nature of the survey data limits our ability to establish temporal associations between purchase behavior and responses to HWLs. Interviews involved convenience samples recruited only in the urban neighborhoods of 2 metropolitan cities; hence, qualitative findings cannot be generalized either to these cities or to other cities and states or rural contexts in India. As most participants purchased loose cigarettes at their last purchase, no comparison could be made with pack purchasers, who could have potentially been exposed more frequently to HWLs.

### Conclusions

Our study provides evidence that the purchase of loose cigarettes reduces exposure to HWLs. Further, those who purchased bidi bundles were less likely to read and look closely at HWLs and think about the harms of bidi smoking and quitting bidi smoking. Strengthening HWL regulations for cigarettes is crucial, but it is equally important to enforce HWL requirements for bidis, given the distinct sociodemographic differences between bidi and cigarette consumers. Research indicates that bidi users are significantly more likely to be male individuals, older adults, and from lower socioeconomic backgrounds [55], while cigarette use is associated with higher socioeconomic status and more affluent lifestyles [31,56]. Interview participants were generally aware of the content of HWLs and intentionally avoided seeing them. Loose cigarette purchases reduced the opportunity to deliver constant reminders about the harmful effects of cigarette smoking owing to reduced exposure. There is a need for strictly implementing and enforcing the prohibition on selling loose cigarettes and adhering to section 7 of COTPA which recommends that all tobacco products should be sold intact in their commercial packs covered with pictorial HWLs. Evidence from observational studies suggests that a loose cigarette ban would reduce cigarette consumption, assist in quitting, prevent smoking initiation, and reduce the chances of relapse [31,57]. We also strongly recommend that efforts be made to increase compliance with the ban on the sale of loose cigarettes in Mumbai, and to assess more rigorously whether HWLs are less effective for loose cigarette and bidi users than for pack and bundle purchasers.

## Abbreviations

COREQ: Consolidated Criteria for Reporting Qualitative Research
COTPA: Cigarettes and Other Tobacco Products Act
HWL: health warning label
STROBE: Strengthening the Reporting of Observational Studies in Epidemiology
TCP: Tobacco Control Policy

## Data Availability

The data sets generated during and/or analyzed during this study are not publicly available but are available from the corresponding author on reasonable request. Researchers interested in using International Tobacco Control Evaluation (ITC) data are required to apply for approval by submitting an International Tobacco Control Data Repository request application and subsequently signing an International Tobacco Control Data Repository Data Usage Agreement. The criteria for data usage approval and the contents of the Data Usage Agreement are described online on the ITC Project website.
